# Evaluation of effective features in the diagnosis of Covid‐19 infection from routine blood tests with multilayer perceptron neural network: A cross‐sectional study

**DOI:** 10.1002/hsr2.1048

**Published:** 2023-01-06

**Authors:** Fatemeh Mohammadi, Leila Dehbozorgi, Hamid Reza Akbari‐Hasanjani, Zahra Joz Abbasalian, Reza Akbari‐Hasanjani, Reza Sabbaghi‐Nadooshan, Hedieh Moradi Tabriz

**Affiliations:** ^1^ Department of Pathology, Sina Clinical‐Research Center Tehran University of Medical Sciences Tehran Iran; ^2^ Department of Electrical Engineering, Central Tehran Branch Islamic Azad University Tehran Iran; ^3^ Department of Analytical Chemistry, School of Chemistry Damghan University Damghan Iran

**Keywords:** blood test, coronavirus, Covid‐19, multilayer perceptron network, neural network

## Abstract

**Background and Aim:**

Coronavirus is an infectious disease that is now known as an epidemic, early and accurate diagnosis helps the patient receive more care. The aim of this study is to investigate Covid‐19 using blood tests and multilayer perceptron neural network and affective factors in improving and preventing Covid‐19.

**Methods:**

This cross‐sectional study was performed on 200 patients referred to Sina Hospital, Tehran, Iran, who were confirmed cases of Covid‐19 by computerized tomography‐scan analysis between 2 March 2020 to 5 April 2020. After verification of lung involvement, blood sampling was done to separate the sera for C‐reactive protein (CRP), magnesium (Mg), lymphocyte percentage, and vitamin D analysis in healthy and unhealthy people. Blood samples from healthy and sick people were applied to the multilayer perceptron network for 70% of the data for training and 30% for testing.

**Result:**

By examining the features, it was found that in patients with Covid‐19, there was a significant relationship between increased CRP and decreased lymphocyte levels, and increased Mg (*p* < 0.01). In these patients, the amount of CRP and Mg in women and the number of lymphocytes and vitamin D in men were significantly higher (*p* < 0.01).

**Conclusion:**

The important advantage of using a multilayer perceptron neural network is to speed up the diagnosis and treatment.

## 1 INTRODUCTION

The severe acute respiratory syndrome coronavirus 2 (SARS‐CoV‐2) epidemic, called Covid‐19, has spread with unprecedented severity and extent around the world. After infecting countries, governments around the world have taken serious measures, such as quarantining hundreds of millions of people in different parts of the world, to combat the spread of Covid‐19 infection.^1^ When an infected person is found, he or she is isolated and treated for recovery.^2^ The patient must be isolated to prevent transmission.

Therefore, it is important to early identify and give treatment to these subsets of patients to reduce the disease severity and improve the outcomes of Covid‐19. Clinical studies demonstrated that altered levels of some blood markers might be linked with the degree of severity and mortality of patients with Covid‐19.^3^ Parameters extracted from the blood test in this article are as follows:

### 1.1 Vitamin D

Vitamin D plays an essential role in the immune system. Vitamin D increases anti‐inflammatory agents. Since the corona outbreak, much research has been published on the importance of vitamin D in coronary control and prevention. Vitamin D can help the immune system fight the virus in several ways.

Vitamin D enhances cellular innate immunity partly through the induction of antimicrobial peptides, including human cathelicidin, LL37, and 1,25‐dihydroxyvitamin D [1,25(OH)2D], defense and modulate cytokine storm of pro‐inflammatory T helper 1 cytokine.^4^ The recent review shows that supplementation with multiple micronutrients plays immune‐supporting roles that modulate the immune function and reduce the risk of infection. In this vein, micronutrients such as vitamins C and D and zinc have the strongest evidence for immune support.^5^

### 1.2 Lymphocytes

Lymphocytes are a type of white blood cell and are an important component of the immune system. These cells are made in the bone marrow and are found in blood and lymph fluid. The immune system is a complex network of cells known as immune cells, including lymphocytes. These cells work together to protect the body against invading agents, such as bacteria, viruses, and cancer cells, all of which threaten the body's health.

Several studies have determined a correlation between the disease and lymphopenia, a condition defined by abnormally low counts of lymphocytes. A closer look at Covid‐19 patients suffering from lymphopenia almost always exhibit significant decreases in T cell counts.^6^

Natural killer cells and cytotoxic T cells are essential in the control of the viral infection. In recent studies (by Huang et al. and Yang et al. in 2020),^7^, ^8^ it was shown that about 85% of severely ill patients with Covid‐19 are suffering the lymphopenia.^9^

### 1.3 C‐reactive protein (CRP)

Measurement of CRP in a medical diagnostic test is a nonspecific test to diagnose rheumatism and arthritis. In the event of microbial infection and infectious agents, the amount of this protein in the blood serum increases and becomes so‐called positive.

Novel coronavirus (2019) seems to increase CRP levels significantly, due to inflammatory reactions and related tissue destruction was also seen in the SARS epidemic in 2002.^10^ Higher concentration indicates more severe disease—linked to lung damage and worse prognosis.^11^

### 1.4 Magnesium (Mg)

Most of the Mg in the body is in the intracellular fluid, and some of it is in the bone and, as a cofactor, regulates the activity of many enzymes. Mg regulates the metabolism and synthesis of carbohydrates, proteins, and nucleic acids. The function of many organs, such as neuromuscular tissue, is also dependent on Mg.

Mg also has anti‐inflammation, anti‐oxidation, antispasm, vasodilation, and neuroprotection. Mg is expected to play an active role in clinical practice in the prevention and treatment of Covid‐19. Therefore, the possibility of the use of Mg supplementation in the prevention and treatment of Covid‐19.^12^

Patients with Covid‐19 symptoms were screened for real‐time reverse transcription polymerase chain reaction on respiratory specimens. In this article, two models of machine learning have been used, the accuracy of diagnosing the disease is between 82% and 86%, and the sensitivity of the learning machine in diagnosing the disease is between 92% and 95%.

In Hastie et al.,^13^ CRP levels were collected, and the diameter of the largest lung lesion was measured. Patients were divided into four groups: mild, moderate, severe, and critical. CRP level and diameter of the largest lung lesion in the moderate group were higher than those in the mild group, higher in the severe group than in the moderate group, and higher in the critical group than in the severe group. Therefore, in the early stages of diagnosis, CRP levels in Covid‐19 are positively and significantly associated with lung lesions and can reflect the severity of the disease.

In Hastie and colleagues,^13^, ^14^, ^15^ the amount of vitamin D was examined and tested. Experiments by Hastie et al.^13^ indicate that there is no potential link between vitamin D concentrations and the risk of Covid‐19.

The conceptual structures and operating systems introduced by Jamshidi et al.^16^ indicate that AI‐based techniques are suitable for coping with and detecting Covid‐19. It includes Covid‐19 diagnostic systems such as recurrent neural networks, long short‐term memory, generative adversarial networks, and extreme learning machines. Geographical issues, high‐risk individuals, and cognition and radiology are the main problems of Covid‐19 and have been studied and discussed in this work.

In this paper, first, with the help of a multilayer perceptron (MLP) neural network, four different parameters of blood testing are examined, the network is trained and tested, and then the accuracy of the network is calculated. With the help of feature selection methods, effective features are distinguished from ineffective ones. This method helps experts and doctors diagnose the disease more quickly and accurately to fight the virus. Success in combating Covid‐19 for its eventual elimination depends on the methods and tools used to detect it early and prevent it from spreading. Achieving the desired goals and achieving the salvation of most lives depends on early detection.

## 2 METHODS

This cross‐sectional study was performed on 200 patients referred to Sina Hospital (Ethics code: IR.TUMS.SINAHOSPITAL.REC.1401.026), Tehran, Iran, who were confirmed cases of Covid‐19 by computerized tomography‐scan analysis between March 2, 2020, and April 5, 2020.

After verification of unilateral or bilateral lung involvement, blood sampling was done and divided into two parts. One part contained a K2EDTA anticoagulant to check lymphocyte percentage, and the other test tube contained a clot activator agent to separate the sera for CRP, Mg, and vitamin D analysis. Measurement of these parameters was performed on fresh samples in the laboratory of Sina Hospital.

To quantify lymphocyte percentage, Sysmex xs 800i (flow cytometry close analyzer) was applied. To measure CRP, Mg, and vitamin D levels, blood samples were centrifuged in 4000*g* for 15 min, and then separated serum was investigated by biochemistry Mindray BS800m (calorimetric close analyzer) to quantify CRP (Lot:38201034; Biorexfars Co. Ltd.) an Mg (Lot:352‐01‐021/3; Biorexfars Co. Ltd.) level in sera and level of vitamin D (Lot:10487UI01; Abbott Co. Ltd.) was measured by autoanalyser Abbott *i* 1000 (chemi luminescence close analyzer). All mentioned devices were calibrated via a specific calibrator, and their accuracy was determined through quality control procedures.

**Figure 1:**
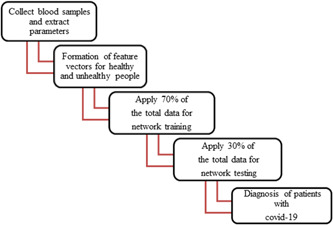
Detection algorithm diagnosis of patients with Covid‐19 from blood test samples

In this study, Covid‐19 infection was diagnosed using an MLP network and features extracted from a blood test along with statistical tests. A block diagram of how to use the MLP network is shown in Figure 1, which generally includes four steps: (1) collecting blood samples from patients and extracting parameters, (2) forming and constructing a feature vector for healthy people and unhealthy, (3) apply 70% of the total data for network training, (4) test the remaining 30% of the data, and (5) diagnose patients with Covid‐19.

### 2.1 MLP network

**Figure 2:**
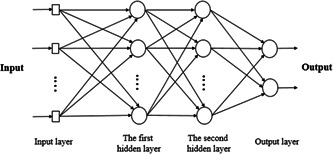
Structure of multilayer perceptron network

An MLP network is a neural network used to segment data. A typical MLP consists of input and output layers, and one or more intermediate layers. First, the network structure is formed, and then the neurons are connected by weights, and the network is trained. Figure 2 shows the structure of this network.^17^ In this paper, the number of MLP input and output nodes is 4 and 2, respectively. The number of nodes in each of the two middle layers is equal to 30. The inputs are multiplied by the weights that are calculated by the network at each node. Then the output of the network is calculated. The network was trained and tested with two classes so that the output contains one of two numbers as follows: If the output becomes zero, it means there is no disease, and if the output becomes one, it means there is a disease.

### 2.2 Diagnosis feature selection

Due to the good performance of UTA,^1^ we use it for feature recognition. In this algorithm, the average of one property is calculated for all samples. The selected property is then substituted in all input vectors with the calculated mean value. The network is then tested with new features. If the detection of the system decreases, those features are effective, but if the results do not change or, conversely, increase, that feature is conceivable and should be removed from the input vector.^1^

To check for more or less system recognition, we can count the number of misdiagnoses that were not in the class and belonged to the class false positive (FP) and the number of misdiagnoses that were in the class and did not belong to the class. Find a false negative (FN) when the entire feature column is still in the training matrix (FP, FN) and then calculate the same parameters for the case where the feature column is deleted (FP1, FN1). Then, calculate the difference between the sums of the above two.

(1) Error=(FP1+FN1)−(FP+FN).

There are three ways to error:

1. Error = 0: The error does not change, so the attribute is not effective.
2. Error > 0: The error increases, so the feature is effective.
3. Error < 0: Error is reduced, so the feature is not effective, but also malicious.

### 2.3 Statistical analysis

Data obtained from sick and healthy individuals were entered into SPSS software ver.21.0 for windows, and their mean was obtained and compared with the mean obtained from healthy individuals. Data distribution was examined using Kolmogorov–Smirnov test. Due to the normal distribution of data in healthy individuals and their abnormal distribution in patients with Covid‐19, a paired sample *t*‐test of two‐tailed was used to evaluate the significance of the differences.

Data were presented as mean ± standard deviation (SD) or mean ± standard error of the mean (SEM). This project uses Pearson's correlation and correlation coefficient between analytics. *p* Value < 0.05 is valued. Statistical analyses were performed by SPSS.

## 3 RESULTS

In this study, the statistical calculation method and artificial neural network have been used. In this study, the feature matrix was applied to the MLP network. Using blood samples collected from Sina Hospital, the characteristics of vitamin D, lymphocytes, CRP, and Mg were first extracted from blood tests. To use the above two modes, the test conditions and the preparation steps and extraction of the characteristics are exactly the same. First, the properties of CRP, Mg, lymphocyte, and vitamin D were extracted from blood samples. The extracted samples were applied to an MLP network for training and testing in the ratio of 70% to 30%, respectively.

The MLP network consists of four layers. The first layer (input) consists of 4 nodes, two middle layers of each of 30 nodes, and the output layer of 2 nodes. The network was trained with 140 samples after 940 replications until the computational error reached close to zero (0.0066 and 0.0071), and then the network was tested with 60 experimental samples.

The demographics (blood test sample [characteristics of CRP, Mg, lymphocyte, and vitamin D]) of sick people with Covid‐19 who participated in this study are shown in Table 1. Table 2 shows the demographic information of people participating in the study related to non‐Covid‐19 people.

**Table 1:** Demographic information of the participants in the study (related to people with Covid‐19)

	Num.	Minimum	Maximum	Means ± SD
Age	200	17.0	87.0	57.4 ± 15.3
CRP	200	2.3	180.2	79.5 ± 50.6
Vitamin D	200	3.7	148.3	24.5 ± 16.1
Lymphocytes	200	1.4	76.2	18.7 ± 10.3
Magnesium	200	1.7	5.5	2.40 ± 0.50

Abbreviation: CRP, C‐reactive protein.

**Table 2:** Demographic information of people participating in the study related to non‐Covid‐19 people

Descriptive statistics					
	*N*	Minimum	Maximum	Mean	SD
H.Chcrp	200	0.45	11.40	5.2148	2.67847
H.Clymph	200	31.20	48.10	39.8860	3.98270
H.C.vitd	200	2.50	113.00	25.4045	17.20392
H.C.mg	200	1.60	2.90	2.1625	0.23733
Valid *N* (listwise)	200				

Table 3 shows the *p*‐value of the correlation between the analytics of the participants in the experiment.

**Table 3:** *p* Value between analytics

*p* Value significant (two‐tailed)	Vitamin D	Lymphocytes	Magnesium	Age	CRP
CRP	0.24	<0.001 (−)	<0.001 (+)	0.07	──
Vitamin D	──	0.28	0.88	0.02 (+)	0.24
Lymphocytes	0.28	──	0.005 (−)	0.18	<0.001 (−)
Magnesium	0.88	0.005 (−)	──	0.72	<0.001 (+)
Sex	<0.001	0.001	0.58	──	<0.001

Abbreviation: CRP, C‐reactive protein.

*p* Value < 0.05 was considered significant in the values associated with the table. The sign (+) indicates direct communication, and (−) indicates inverse communication.

According to Table 3, in patients with Covid‐19, there was a significant relationship between increased CRP and decreased lymphocyte levels, and increased Mg (*p* < 0.01). With the increasing age of people with Covid‐19, the amount of vitamin D increases (*p* < 0.05). In these patients, the amount of CRP and Mg in women and the number of lymphocytes and vitamin D in men were significantly higher (*p* < 0.01).

**Figure 3:**
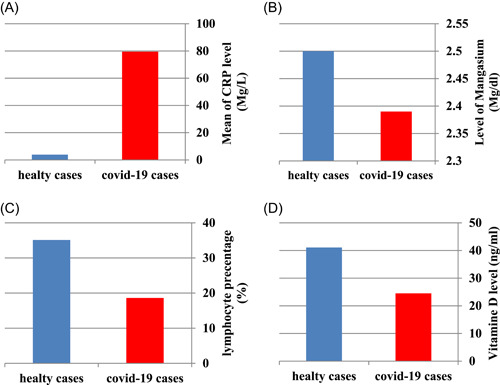
Difference between healthy and sick individuals for different clinical values (A) difference CRP, (B) difference Mg, (C) difference lymphocyte, and (D) difference vitamin D. CRP, C‐reactive protein; MP, magnesium.

The mean difference between healthy and sick individuals for different clinical values is shown using Figure 3.

The difference in values obtained for vitamin D in healthy individuals and Covid‐19 individuals was not significant, but the values related to CRP, Mg, and lymphocyte percentage in these two groups with a *p*‐value of 0.0 were considered significant. Table 4 shows the differences in this Mg, CRP, lymphocyte, and vitamin D parameters between healthy individuals and Covid‐19 individuals.

**Table 4:** Differences in this Mg, CRP, lymphocyte, and vitamin D parameters between healthy individuals and Covid‐19 individuals

	Standard deviation	Standard error of the mean	95% Confidence interval of the difference		*t*	df	Significant (two‐tailed)
			Lower	upper			
Mg	0.50672	0.03864	−0.30185	−0.14931	−5.838	171	<0.001
CRP	50.67761	3.63844	67.11755	81.46997	20.419	193	<0.001
Lymphocyte	11.16231	0.79128	−22.76940	−19.64859	−26.804	198	<0.001
Vitamin D	23.23582	1.64714	−4.15222	2.34418	−0.549	198	0.584

Abbreviations: CRP, C‐reactive protein; MP, magnesium.

**Figure 4:**
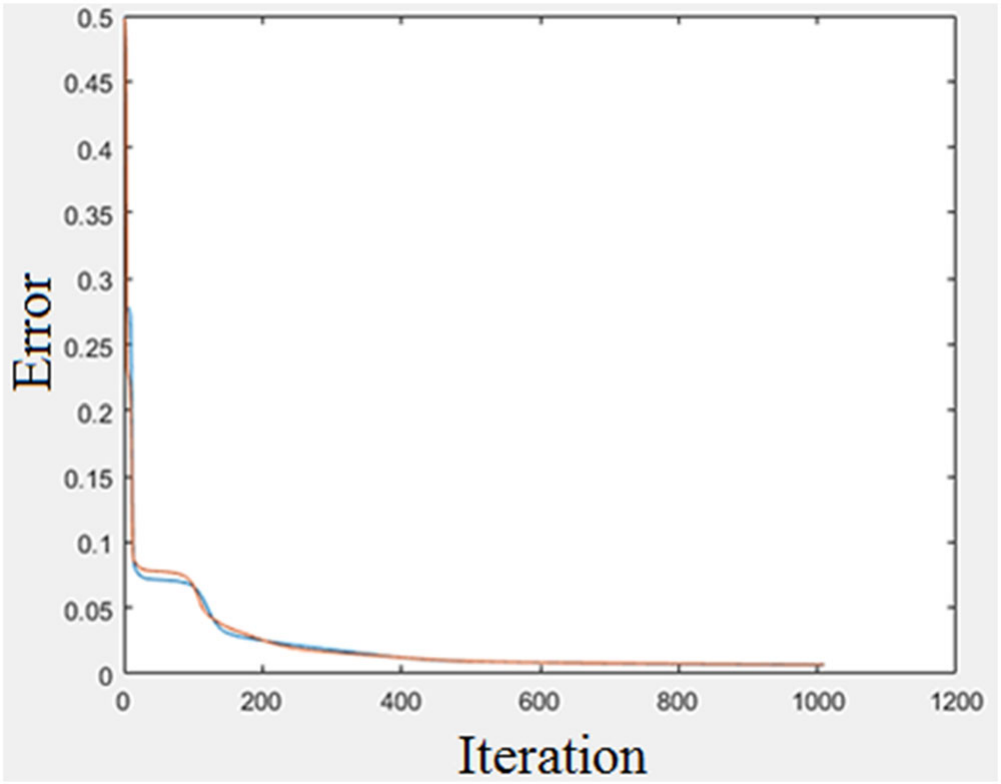
Difference between multilayer perceptron network output and actual output after more than 940 iterations (error decreases)

The perceptron network was used with 99.1667% accuracy to diagnose people with Covid‐19. The value of network sensitivity is 0.9833, and network specificity is 1. These results are shown in Table 5. Figure 4 shows the difference between the output of an MLP network and the actual output after 940 iterations. As can be seen, the error is decreasing.

**Table 5:** Results of multilayer perceptron network performance for the diagnosis of Covid‐19

Accuracy	99.1667	
Sensitivity	0.9833	
specificity	1	
Error	0.0156	0.0109

The results show that the MLP network can detect Covid‐19 individuals from blood samples with relatively good accuracy.

### 3.1 Extract properties

After completing the training and testing phase by the multilayer perceptron classifier, using the UTA algorithm,^1^ and the effective features in the diagnosis of Covid‐19 patients by the MLP network were selected. According to formula (1), if the error is zero, the property is not effective. If it is greater than zero, it is effective, and if it is less than zero, it is destructive.

Table 6 shows that the error is positive in the first and last two cases, that is, the CRP parameter with the highest error is 59, the most effective parameter, and then the lymphocyte parameter with an error value of 18 is affected in the second order. Mg and vitamin D parameters are not effective properties.

**Table 6:** Feature selection results

	Error 1	FP1	FN1	TP1	TN1
CRP	59	60	60	0	0
Mg	0	0	1	59	60
Vitamin D	0	0	1	59	60
Lymphocyte	18	18	1	59	42

Abbreviations: CRP, C‐reactive protein; FN, false negative; FP, false positive; MP, magnesium; TN, true negative; TP, true positive.

## 4 CONCLUSION

**Figure 5:**
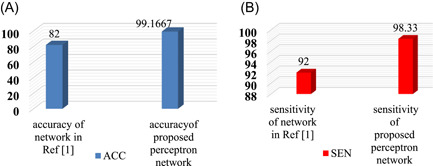
Comparison of accuracy characteristics (ACC) and sensitivity (SEN) of the proposed multilayer perceptron network in comparison with Brinati et al.^1^; (A) comparison of accuracy, (B) sensitivity comparison.

The present study shows that the characteristics of CRP and lymphocytes extracted from blood tests can be used for a more accurate and early diagnosis of people with Covid‐19. The proposed method uses a new feature matrix that distinguishes infected people from healthy individuals with acceptable accuracy of 99.1667% with the help of an MLP network. The features used in this algorithm could play a good role in diagnosing the disease. Figure 5 the results of the proposed MLP network are compared with the results. The accuracy and sensitivity parameter of the proposed network has a better performance than Brinati et al.^1^ The purpose of this research is not only to diagnose the disease. Rather, after the diagnosis, the effective features in the diagnosis of the disease are introduced. The results show that among all four input features, two features are more effective in diagnosing the disease. The CRP and the lymphocyte parameters are effective features. Reducing the dimensions of the input matrix, which contains only the effective features, helps to diagnose the disease faster.

In a study by Lagadinou et al., all people who came to Patras Hospital with a positive test for Covid‐19 were evaluated for laboratory factors. It was shown that factors such as neutrophil to lymphocyte ratio, lactate dehydogenase (LDH), D‐dimers, CRP, fibrinogen, and ferritin were associated with the disease, and the team noted that based on the results, they will be able to diagnose the severity of the disease in the future from the first examination. These results are confirmed by the results of computational analyzes performed in this study, and the severity and prognosis of the disease in the future can be determined from clinical factors.^18^

In a study by An et al., various clinical factors such as CRP, erythrocyte sedimentation rate, LDH, and lymphocyte counts were examined in 64 patients with Quid. Significant changes in these factors were obtained in these individuals, and it was also observed that after treatment, these values approached their normal values. Also, in this study, body mass index was evaluated, and it was observed that a higher rate is associated with longer hospital stays. Although the number of subjects in this study was small, the results were confirmed by the results of the present study as well as other studies.^19^

A study by Gayda et al. found an increase in CRP levels in people with Covid‐19, whereas a decrease in lymphocyte counts was observed. A negative correlation was found between age and lymphocyte count, and a direct correlation was observed between age and lymphocyte count. However, in the present study, no significant relationship was observed between age and serum CRP level and the percentage of blood lymphocytes.^20^

In another study by M. Wang et al., various parameters such as CRP, lymphocyte, cardiac troponin I, D‐dimer, procalcitonin, and interleukin 6 were examined, and an increase in all parameters was associated with an increase in disease severity, while a decrease in lymphocyte levels with an increase. The severity of the disease has been associated. In the present study, a significant relationship was observed between CRP levels and lymphocyte levels, which can actually confirm this.^21^

In the present study, a significant and direct relationship was found between the amount of CRP and the level of Mg ions. Also, Mg ions had a significant inverse relationship with the level of blood lymphocytes. This can be due to the role of Mg ions in the functioning of the immune system and inflammation.

According to a study by Meltzer et al.,^22^ examining the history of vitamin D levels in the blood of 489 people tested for coronavirus, concluded that a decrease in the amount of vitamin D in the blood of individuals was associated with an increased risk of developing a virus.^23^ In the present study, the level of vitamin D in people with coronavirus was examined, and therefore, the probability of infection cannot be estimated based on the obtained results. Since in this study, no significant relationship was found between the level of vitamin D and CRP and lymphocytes, so the severity of the disease cannot be interpreted in combination with this vitamin.

To better interpret clinical data and their relationship with the prognosis and chances of people with coronary heart disease, it is better to design studies in which cross‐sectional and at the beginning of the disease, during the disease, and during recovery. It is also possible to obtain better information about the severity and severity of the disease in relation to clinical parameters by following patients and comparing clinical parameters in people in whom the disease leads to hospitalization in intensive care units or death.

## 5 SUGGESTION

It is recommended that more blood samples be collected as soon as possible, and if possible, images of their lungs are processed using a neural network and use both features for accurate and early diagnosis.

## AUTHOR CONTRIBUTIONS

**Fatemeh Mohammadi**: Formal analysis. **Leila Dehbozorgi**: Data curation; software. **Hamid Reza Akbari‐Hasanjani**: Software; supervision; visualization; writing – review & editing. **Zahra Joz Abbasalian**: Formal analysis; writing – original draft. **Reza Akbari‐Hasanjani**: Data curation; software; writing – original draft. **Reza Sabbaghi‐Nadooshan**: Methodology; software; writing – review & editing. **Hedieh Moradi Tabriz**: Writing – review & editing.

## CONFLICT OF INTEREST

The authors declare no conflict of interest.

## ETHICS STATEMENT

This study was approved by the ethics committee of the Tehran University of Medical Sciences (Sina Clinical‐Research Center) in Tehran, Iran, also informed consent was obtained for those eligible to enter the study.

## TRANSPARENCY STATEMENT

The lead author Hamid Reza Akbari‐Hasanjani affirms that this manuscript is an honest, accurate, and transparent account of the study being reported; that no important aspects of the study have been omitted; and that any discrepancies from the study as planned (and, if relevant, registered) have been explained.

## Data Availability

The data are not publicly available due to privacy or ethical restrictions.
